# *Histoplasma capsulatum *proteome response to decreased iron availability

**DOI:** 10.1186/1477-5956-6-36

**Published:** 2008-12-24

**Authors:** Michael S Winters, Daniel S Spellman, Qilin Chan, Francisco J Gomez, Margarita Hernandez, Brittany Catron, Alan G Smulian, Thomas A Neubert, George S Deepe

**Affiliations:** 1 Division of Infectious Diseases, University of Cincinnati College of Medicine, Cincinnati, OH 45267, USA; 2 Department of Pharmacology and Kimmel Center for Biology and Medicine at the Skirball Institute, New York University School of Medicine, New York, NY 10016, USA; 3 Department of Chemistry, University of Cincinnati, Cincinnati, OH 45221-0172, USA; 4 Veterans Affairs Hospital, University of Cincinnati College of Medicine, Cincinnati, OH 45267, USA; 5 Department of Biology, University of Texas at San Antonio, One UTSA Circle, San Antonio, TX 78249-006, USA

## Abstract

**Background:**

A fundamental pathogenic feature of the fungus *Histoplasma capsulatum *is its ability to evade innate and adaptive immune defenses. Once ingested by macrophages the organism is faced with several hostile environmental conditions including iron limitation. *H. capsulatum *can establish a persistent state within the macrophage. A gap in knowledge exists because the identities and number of proteins regulated by the organism under host conditions has yet to be defined. Lack of such knowledge is an important problem because until these proteins are identified it is unlikely that they can be targeted as new and innovative treatment for histoplasmosis.

**Results:**

To investigate the proteomic response by *H. capsulatum *to decreasing iron availability we have created *H. capsulatum *protein/genomic databases compatible with current mass spectrometric (MS) search engines. Databases were assembled from the *H. capsulatum *G217B strain genome using gene prediction programs and expressed sequence tag (EST) libraries. Searching these databases with MS data generated from two dimensional (2D) in-gel digestions of proteins resulted in over 50% more proteins identified compared to searching the publicly available fungal databases alone. Using 2D gel electrophoresis combined with statistical analysis we discovered 42 *H. capsulatum *proteins whose abundance was significantly modulated when iron concentrations were lowered. Altered proteins were identified by mass spectrometry and database searching to be involved in glycolysis, the tricarboxylic acid cycle, lysine metabolism, protein synthesis, and one protein sequence whose function was unknown.

**Conclusion:**

We have created a bioinformatics platform for *H. capsulatum *and demonstrated the utility of a proteomic approach by identifying a shift in metabolism the organism utilizes to cope with the hostile conditions provided by the host. We have shown that enzyme transcripts regulated by other fungal pathogens in response to lowering iron availability are also regulated in *H. capsulatum *at the protein level. We also identified *H. capsulatum *proteins sensitive to iron level reductions which have yet to be connected to iron availability in other pathogens. These data also indicate the complexity of the response by *H. capsulatum *to nutritional deprivation. Finally, we demonstrate the importance of a strain specific gene/protein database for *H. capsulatum *proteomic analysis.

## Background

*Histoplasma capsulatum *is a dimorphic fungus and the etiological agent of histoplasmosis. The fungus is endemic to the Midwestern and southeastern United States. *H. capsulatum *can be a life-threatening infection for individuals suffering from immune defects associated with AIDS and for those receiving immunosuppressive pharmaceuticals to combat malignancy, graft rejection, and autoimmunity. More recently, antagonists to tumor necrosis factor-α have been identified as a risk factor for the development of disseminated histoplasmosis [1-5].

*H. capsulatum *survives in the mammalian host by evading both the innate and adaptive immune responses. *H. capsulatum *is confronted by several phagocytic cell populations including immature dendritic cells, neutrophils, and macrophages. Among them, the last is the only professional phagocytic cell population in which *H. capsulatum *can replicate freely [6]. Intracellularly, *H. capsulatum *must confront and adapt to phagolysosomal fusion, nitrosative stresses, low nutrient availability, and low pH [7-12].

Two-dimensional electrophoresis in combination with mass spectrometry has become a powerful tool for studying the proteome of a number of intracellular pathogens [13-19]. There have been limited microarray and proteomic analyses of *H. capsulatum *[20-22]. For fungi such as *H. capsulatum*, such information is nascent. Therefore we have utilized the publicly available *H. capsulatum *G217B genome and transcripts developed for microarray analysis to construct protein/genomic databases compatible with mass spectrometry (MS) data search engines. We have tested the utility of these databases and proteomics for *H. capsulatum *by studying the response under iron-limiting conditions.

Iron is a major nutrient for growth of *H. capsulatum *both *in vitro *and *in vivo *[23-25]. Sequestration of iron by interferon gamma (IFN-γ) activated macrophages acts as a host defense mechanism against *H. capsulatum *[26]. The influence of iron availability on metabolic processes in *H. capsulatum *has only begun to be addressed. Recently a connection between iron availability and lipid metabolism was shown in *H. capsulatum *as iron-related alterations influenced the content of triacylglycerol and free fatty acids [27]. We have utilized 2D gel electrophoresis, statistical analysis, MS, and bioinformatics to identify *H. capsulatum *proteins sensitive to lowering iron availability. Using this platform, we discovered 42 *H. capsulatum *proteins whose abundance was altered when grown in the presence of iron scavenger apo-transferrin. Proteins found to be sensitive to decreasing iron levels suggest a requirement for *H. capsulatum *to mediate specific metabolic functions in order to cope with changes in the environment the organism most likely encounters in the host.

## Results and Discussion

### Proteomic platform development

**Figure 1:**
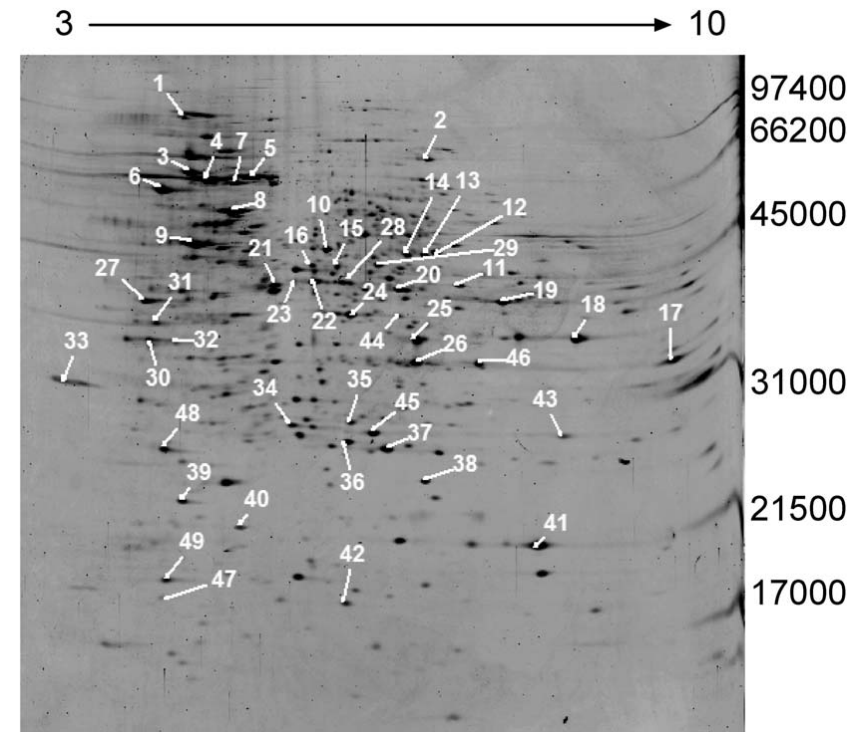
**2D-gel analysis of proteins from *H. capsulatum *following 3-day growth**. Identified spots are numbered and listed in table 1. The gel was loaded with 50 μg of total protein and stained with SYPRO Ruby fluorescent dye. The pH gradient is labeled on the top of the gel and molecular weight markers in Daltons are labeled on the right.

In order to set-up a platform for analyzing the *H. capsulatum *proteome, the optimal conditions for protein extraction from *H. capsulatum *yeast had to be determined. Several commercial lysis buffers were tested in our laboratory before selecting a buffer which provided the highest protein yield. The lysis buffer which yielded the highest protein yield was composed of 9 M urea, 2% CHAPS, 1% DTT, and 10 mM protease inhibitor. Figure 1 represents the 2D gel profile of proteins from *H. capsulatum *grown in liquid culture for 3 days and lysed using the aforementioned buffer. We consistently detected ~1500 protein spots.

We searched matrix assisted laser desorption/ionization time-of-flight (MALDI-TOF) mass spectrometer (MS) and nanoflow liquid chromatography (LC)-MS/MS data obtained from 2D in-gel digests of protein spots labeled in figure 1 against all the MASCOT searchable publicly available eukaryotic databases. Protein spots were selected based on intensity in order to test the quality of the different gene/protein databases to generate the most protein identifications. Following download and MASCOT modifications we searched the G186 database available at the Broad Institutes Fungal Genome Initiative. . Searching the public available databases and Broad yielded identifications for 34 out of 49 individual protein in-gel digests. In order to increase our protein identifications, a gene/protein database specific for the G217B *H. capsulatum *strain and compatible with MS search engines was constructed using a combination of different gene finding programs.

Database 1, consisting of ~9348 sequences, was constructed using Fgenesh and parameters setup by Softberry . Fgenesh finds genes based on intrinsic characteristics of coding sequences such as codon usage and consensus splice sites [28]. Database 2 was assembled using the publicly available EST libraries of both mycelial and yeast phases, and assembled into full gene sequences with the aid of the CAP3 genome assembly program [29] yielding a database containing 6,233 gene sequences. Database 3 was originally assembled by Washington University St. Louis Genome Sequencing Center for microarray analysis using a combination of different gene finding programs which include Fgenesh, EAnnot, GeneWise, and SNAP [28,30-32]. Database 3 contains a total of 14,506 predicted gene sequences.

MS data acquired from in-gel digests of proteins excised from the gel in figure 1 were searched against the 3 in-house strain specific databases leading to the identification of all in-gel digests searched (table 1). Searching the data against the publicly available eukaryotic databases and Broad sequences led to the identification of 34 proteins. Fifteen of these proteins would not have been identifiable without the strain specific databases. These fifteen proteins are marked by an * in table 1. This illustrates the importance of having gene/protein sequence databases that are organism and strain-specific when conducting proteomic analyses.

**Table 1:** List of proteins identified from the 2D gel shown in figure 1 following in-gel tryptic digestion, MALDI-MS analysis, and database searching.

Spot #	BLAST ID	# Peptides Matched/#Searched	% Protein Coverage	Accession #	MASCOT Score (p < .05)
Spot 1*	predicted protein [Ajellomyces capsulatus NAm1].	8/17	12%	XP_001538505	71
Spot 2	aconitate hydratase, mitochondrial precursor [Ajellomyces capsulatus NAm1].	13/29	14%	XP_001539799	107
Spot 3	heat shock 70 kDa protein C precursor [Ajellomyces capsulatus NAm1].	12/17	21%	XP_001538200	150
Spot 4	heat shock protein 70 [Paracoccidioides brasiliensis].	12/16	17%	AAK66771	162
Spot 5 (A10)	70 kDa heat shock protein [Paracoccidioides brasiliensis].	12/26	20%	AAP05987	125
Spot 6*	protein disulfide-isomerase precursor [Ajellomyces capsulatus NAm1].	6/9	12%	XP_001541532	77
Spot 7	heat shock 70 kDa protein [Ajellomyces capsulatus NAm1].	7/10	14%	XP_001543760	90
Spot 8	heat shock protein 60, mitochondrial precursor [Ajellomyces capsulatus NAm1].	18/26	34%	XP_001539356	242
Spot 9	ATP synthase beta chain, mitochondrial precursor [Ajellomyces capsulatus NAm1].	13/24	37%	XP_001539339	159
Spot 10 (A21)	enolase [Paracoccidioides brasiliensis].	13/21	37%	ABQ45367	172
Spot 11	ubiquinol-cytochrome C reductase complex core protein 2, putative [Aspergillus fumigatus Af293].	9/22	25%	XP_001539391	103
Spot 12	dihydrolipoamide dehydrogenase [Ajellomyces capsulatus NAm1].	8/11	17%	XP_747922	119
Spot 13*	fumarate hydratase class II [Aspergillus terreus NIH2624].	8/20	16%	XP_001209891	91
Spot 14*(A29)	saccharopine dehydrogenase [Ajellomyces capsulatus NAm1].	6/16	14%	XP_001544528	64
Spot 15	elongation factor Tu, mitochondrial precursor [Aspergillus terreus NIH2624].	12/21	38%	XP_001210502	170
Spot 16	pyruvate dehydrogenase E1 component alpha subunit, mitochondrial precursor [Ajellomyces capsulatus NAm1].	9/18	21%	XP_001544313	106
Spot 17 (A38)	malate dehydrogenase [Ajellomyces capsulatus NAm1].	7/17	21%	XP_001541871	86
Spot 18	glyceraldehyde-3-phosphate dehydrogenase [Ajellomyces capsulatus].	8/16	35%	AAG33368	112
Spot 19	ketol-acid reductoisomerase, mitochondrial precursor [Ajellomyces capsulatus NAm1].	20/37	46%	XP_001536226	258
Spot 20	mannitol-1-phosphate dehydrogenase [Paracoccidioides brasiliensis].	16/22	44%	AAO47089	234
Spot 21	conserved hypothetical protein [Ajellomyces capsulatus NAm1].	9/18	25%	XP_001544536	118
Spot 22	peptidyl-prolyl cis-trans isomerase [Ajellomyces capsulatus NAm1].	14/30	35%	XP_001536169	170
Spot 23	peptidyl-prolyl cis-trans isomerase [Aspergillus fumigatus Af293].	14/31	27%	XP_001536169	100
Spot 24*	fructose 1,6-biphosphate aldolase 1 [Paracoccidioides brasiliensis].	4/7	8%	AAL34519	62
Spot 25*	RACK1-like protein [Paracoccidioides brasiliensis].	6/16	17%	ABA33785	76
Spot 26*	cytochrome c peroxidase Ccp1 [Aspergillus fumigatus Af293].	9/28	25%	XP_751914	102
Spot 27*	protein disulfide-isomerase precursor [Ajellomyces capsulatus NAm1].	5/12	7%	XP_001541532	68
Spot 28	gene encoding Histoplasma capsulatum predicted protein (2481 nt)	5/12	5%	HCAG_01143	65
Spot 29*	mRNA binding post-transcriptional regulator (Csx1) [Aspergillus fumigatus Af293].	5/13	16%	XP_746709	66
Spot 30*	gene encoding Histoplasma capsulatum predicted protein (2328 nt)	5/16	10%	HCAG_06550	68
Spot 31*	hypothetical protein RUMOBE_03015 [Ruminococcus obeum ATCC 29174]	5/9	9%	ZP_01965283	74
Spot 32	HCAG_04492	4/7	7%	-	60
Spot 33	Hsp90 binding co-chaperone (Sba1) [Aspergillus fumigatus Af293].	6/18	19%	XP_753264	72
Spot 34	thiol-specific antioxidant [Ajellomyces capsulatus NAm1].	10/21	38%	XP_001538605	142
Spot 35*	triosephosphate isomerase [Aspergillus clavatus NRRL 1].	7/19	33%	XP_001274623	103
Spot 36	30 kDa heat shock protein [Ajellomyces capsulatus NAm1].	10/28	31%	XP_001540271	125
Spot 37	superoxide dismutase, mitochondrial precursor [Ajellomyces capsulatus NAm1].	5/16	21%	XP_001541351	78
Spot 38	ATP synthase D chain, mitochondrial [Ajellomyces capsulatus NAm1].	7/19	31%	XP_001539631	102
Spot 39	tropomyosin, putative [Neosartorya fischeri NRRL 181].	6/12	27%	XP_001261343	98
Spot 40*	protein of unknown function DUF181 [Methylobacterium radiotolerans JCM 2831].	5/9	14%	YP_001756253	76
Spot 41	peptidyl-prolyl cis-trans isomerase (cyclophilin) [Ajellomyces capsulatus NAm1].	7/24	26%	XP_001540645	91
Spot 42	putative cytochrome c oxidase subunit VIa [Paracoccidioides brasiliensis].	5/14	13%	AAT77146	76
Spot 43	woronin body major protein [Ajellomyces capsulatus NAm1].	4/8	15%	XP_001543637	63
Spot 44 (A5)	HCAG_04147 (Hypothetical Protein)	5/10	9%	-	66
Spot 45*(A42)	asparaginyl-tRNA synthetase [Flavobacterium sp. MED217].	6/18	16%	ZP_01059347	65
Spot 46	ATPase associated with various cellular activities AAA_3 [Shewanella woodyi ATCC 51908]	6/16	11%	YP_001761394	67
Spot 47*	cystathionine beta-lyase [Rhizobium etli CFN 42].	6/14	13%	YP_469412	62
Spot 48	heat shock protein 70 [Paracoccidioides brasiliensis].	7/11	12%	AAK66771	120
Spot 49	hypothetical protein NCU07591 [Neurospora crassa OR74A].	4/7	7%	XP_962971	64

**Figure 2:**
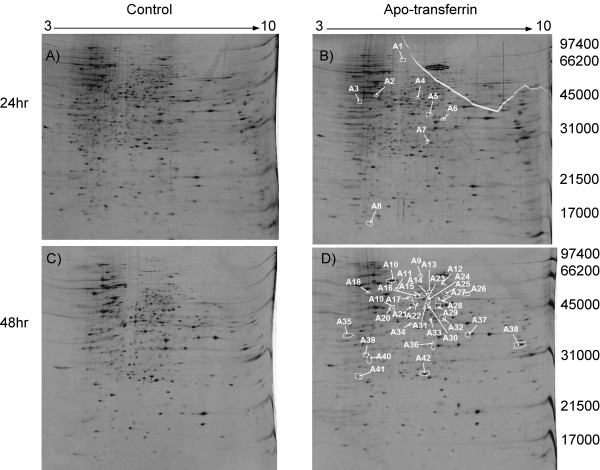
**2D-gel analysis of *Histoplasma capsulatum *proteins **following A) 3-day growth in liquid culture. B) 2-day growth in liquid culture followed by 24 hr with growth media containing 5 μM apo-transferrin. Protein spot circled in black is apo-transferrin C) 4-day growth in liquid culture D) 2-day growth followed by 48 hr with growth media containing 5 μM apo-transferrin. Proteins selected as statistically different in their protein abundance between control and apo-transferrin treated are circled and numbered in white by the SameSpots sofware. The pH gradient is labeled on the top of the gel and molecular weight markers are labeled in Daltons on the right. Gels were loaded with 50 μg of total protein and stained with SYPRO Ruby fluorescent dye.

Numbers in parentheses correspond to protein ID in figure 2.

The protein identifications (BLAST IDs) listed in table 1 are the best homology and functional matches following a BLAST search of the MS matched protein sequence in our custom databases against the NCBInr sequence database. If a protein sequence matched with a hypothetical sequence in the BLAST search, the next best protein BLAST sequence match with annotated functionality was listed. Forty percent of proteins detected are involved in energy or metabolism; 30% belonged to the cell fate and rescue functional cluster. The most intensely SYPRO stained protein bands were heat shock proteins (HSP)s. In addition, MS data from spots 32 and 44 (Figure 1) matched two protein sequences in the Broad 186 strain databases only, neither of which had homology to any other protein sequence following BLAST searching.

Our mass fingerprinting (MFP) analysis was validated using a more sensitive and structurally informative technique, LC-MS/MS analysis. We analyzed 3 digested proteins (Figure 1 spots; 6, 8, and 41) by MFP and LC-MS/MS (table 1) and all 3 matched with their respective protein using both MFP and LC-MS/MS. This included peptide sequence information for 14 fragments from spot 6, six fragments from spot 8, and 3 from spot 41.

Databases were further validated using MS data acquired from in-gel digestion of HSP 60. MS data from in-gel tryptic digested HSP 60 matched exceptionally well with both public and in-house constructed databases. We matched 18 MS generated molecular weights with 18 amino acid sequences to our in-house constructed databases for HSP 60. Using the same MS data to search the publicly available fungal databases using MASCOT, we matched 13 peptides. This also shows the specificity of our databases for *H. capsulatum*, in that we were able to identify 5 additional peptides unique to *H. capsulatum *G217B, HSP 60 (data not shown).

### Iron depletion and 2D gel analysis

Because iron utilization is crucial for successful *H. capsulatum *infection and limiting iron to the organism is believed to be a strategy used by the host, we analyzed *H. capsulatum's *protein response to decreases in iron availability. *H. capsulatum *requires iron for survival inside the host as chelation of iron inhibits fungal growth within mouse and human macrophages [23,26]. In mammalian hosts iron is bound to transferrin and lactoferrin or stored in ferritin. It has been proposed that *H. capsulatum *may acquire iron from transferrin by modulating pH [23] or by secreting siderophores [33]. Transcripts of genes coding for siderephore biosynthesis are upregulated under low iron conditions, and one gene, SID1, is involved in host colonization [34]. Another iron acquisition strategy is through the secretion of ferric reducing compounds [25,35]. Recently it has been shown that as *H. capsulatum *has a preference for ferrous iron [27], and a possible mechanism for *H. capsulatum *iron acquisition is through the ferric reductant activity of a γ-glutamyltransferase [36].

To create an environment more closely resembling that of the mammalian host, we used apo-transferrin to reduce the concentration of free iron in the medium in which *H. capsulatum *was grown by ~90%. Following 48 hr growth in culture *H. capsulatum *was switched to a media containing 5 μM apo-transferrin for 24 hr and 48 hr. Following lysis of cells, 2D gel analysis was performed as shown in figure 2. The 2D gels were analyzed using SameSpots software developed by Nonlinear Dynamics. SameSpots detected and matched 3381 and 3968 protein spots for 24 and 48 groups respectively. A total of 146 and 154 spots were selected as significantly altered in abundance following 24 hr and 48 hr groups respectively. After manual inspection and removal of streaks, areas around streaks, dirt, etc. approximately 1500 proteins spots were accepted as valid protein spots. Of these 1500 spots, a total of 42 proteins (~3.0%) were accepted as significantly altered (p < 0.05) in abundance following *H. capsulatum *growth in the presence of apo-transferrin for 24 hr and 48 hr.

Proteins with statistically significant spot volume changes are circled in white, numbered in Figures 2B and 2D. The 42 proteins circled in white represent fold changes in a range of 1.3–2.8, and p values < .05. Spot volume ratios of all proteins detected and matched using SameSpots were subjected to a global one-way ANOVA statistical test. ANOVA was used to define a subset of individual proteins that exhibited statistically significant abundance changes (normalized volume ratios) as one-way ANOVA analysis is designed to test differences between means in multiple sample groups. Twenty four and 48 hr groups were analyzed separately.

### Proteins sensitive to changes in iron availability

Seven *H. capsulatum *proteins were more abundant while 35 proteins were less abundant following growth in iron-poor (apo-transferrin) medium. A list of these proteins and their corresponding fold changes and p values can be found in the additional file 1. Protein spots identified by MS are labeled A for apo-transferrin in table 1. Protein sequences with homology to enolase (spot A21 Figure 2D), malate dehydrogenase (spot A38 Figure 2D), saccharopine dehydrogenase (spot A29 Figure 2D), HSP 70 (spot A10 Figure 2D), asparaginyl-tRNA synthetase (spot A42 Figure 2D), and an uncharacterized protein with an amino acid sequence unique to *H. capsulatum *(spot A5 Figure 2B) were among the 35 proteins less abundant following *H. capsulatum *growth in apo-transferrin medium. Fold changes and p values calculated by the SameSpots software were as follows; enolase (-1.4 fold change/p value = .0193), malate dehydrogenase (-1.4/.02920), saccharopine dehydrogenase (-1.5/.0377), HSP 70 (-1.4/.0227), asparaginyl-tRNA synthetase (-1.4/.0422), and an uncharacterized protein unique to *H. capsulatum *(-1.4/.0293). This protein was less abundant during the first 24 hr incubation with apo-transferrin and returns to baseline another 24 hr later (Figure 2D, A5).

**Figure 3:**
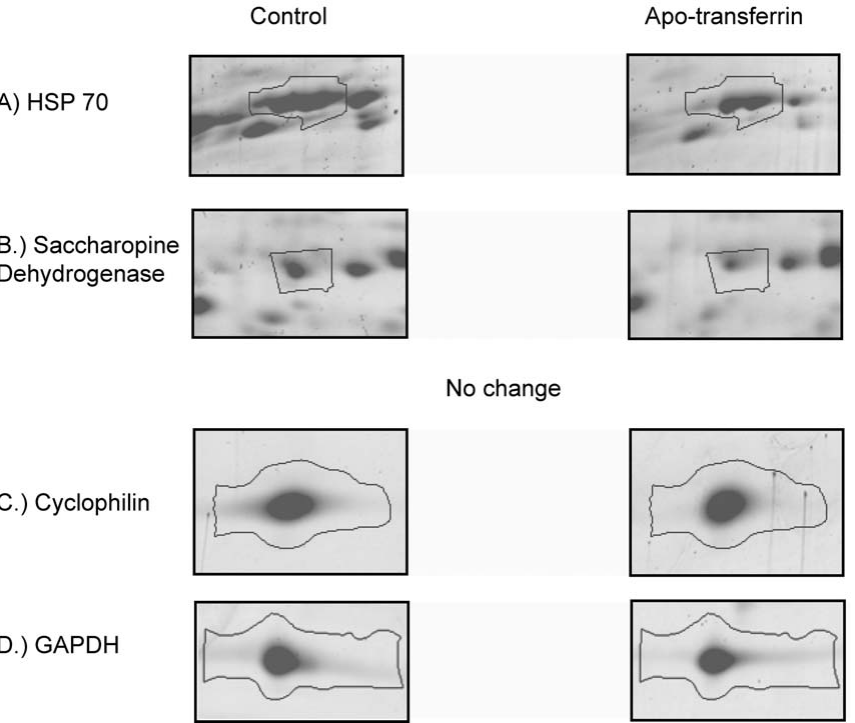
**Magnified regions of the 2D gels shown in figures 2c and 2d**. A.) Protein band corresponds with HSP70 (A10) B.) Protein band corresponds to saccharopine dehydrogenase (A29) C.) Protein bands cyclophilin and GAPDH did not change significantly in their abundance following apo-transferrin treatment. Protein band outlines were created by the SameSpots software.

Magnified regions from the 2D gels for proteins with sequence homology to HSP 70, saccharopine dehydrogenase, cyclophilin and GAPDH are shown in Figure 3. Figure 3A depicts saccharopine dehydrogenase, labeled A29 in Figure 2D. The calculated fold change and p value was -1.5 and 0.0377 respectively. HSP 70, spot A10 in Figure 2D, was also statistically less abundant in iron replete medium and the fold change was -1.4 (p = 0.0227). Peptidyl-prolyl cis-trans isomerase (cyclophilin) and GAPDH were not statistically different in the presence or absence of apo-transferrin.

Enolase and malate dehydrogenase are involved in cellular respiration steps of the tricarboylic acid (TCA) or Krebs cycle and the last step of glycolysis before entering the TCA cycle respectively. Enolase, malate dehydrogenase, and other transcripts involved in the TCA cycle were also less abundant following microarray analysis of *C. albicans, C. neoformans*, and *Escherichia coli *under iron depletion conditions [37-39]. Fold changes of 1.56 and 1.87 for enolase and malate dehydrogenase transcripts respectively detected by microarray analysis of *C. neoformans *[38] under reduced iron availability was similar to those observed in our analysis. Isolating *H. capasulatum *following ingestion from IFN-γ activated macrophages we found that malate dehydrogenase was also significantly altered in abundance (fold change of 5.6, p = 0.0153) compared to the organism grown under the same conditions in culture (additional file 2). When faced with limitation of iron, yeasts will not only increase their iron uptake and their mobilization of stored iron, but also adjust their metabolism to more efficiently use the iron that is available [40]. The availability of iron in *H. capsulatum *has also been shown to affect the amount of triacylglycerols implicating a potential role of iron in lipid metabolism[27].

In addition to proteins involved in cellular respiration, we observed HSP 70 less abundant in *H. capsulatum *when grown in an iron deficient environment. HSP 70 is involved in the biogenesis of iron cluster proteins. Again our results are consistent with what was observed following microarray analysis of transcripts which code these proteins in *C. ablicans *following iron depletion [37]. HSPs may chaperone Fe-S proteins or may be involved in the transfer or iron centres to recipient proteins thus their productivity may decrease when iron levels are lowered.

We also discovered proteins with sequence homology to saccharopine dehydrogenase and asparaginyl-tRNA synthetase whose abundance was decreased when *H. capsulatum's *growth medium iron concentrations were reduced. Saccharopine dehydrogenase is an enzyme involved in lysine metabolism while tRNA synthetase enzyme is required for protein synthesis by catalyzing the specific attachment of asparagine to its cognate tRNA. Out of ~1500 proteins profiled in our model we observed ~2.0% reduction in protein abundance when *H. capsulatum *was incubated with apo-transferrin for 48 hr. Taken together these data could suggest a global reduction in protein synthesis by *H. capsulatum *when confronted with reductions in iron availability. To the authors knowledge neither asparaginyl-tRNA synthetase or saccharopine dehydrogenase have been connected to any other organism's response to lowering iron levels.

### Western blot analysis

**Figure 4:**
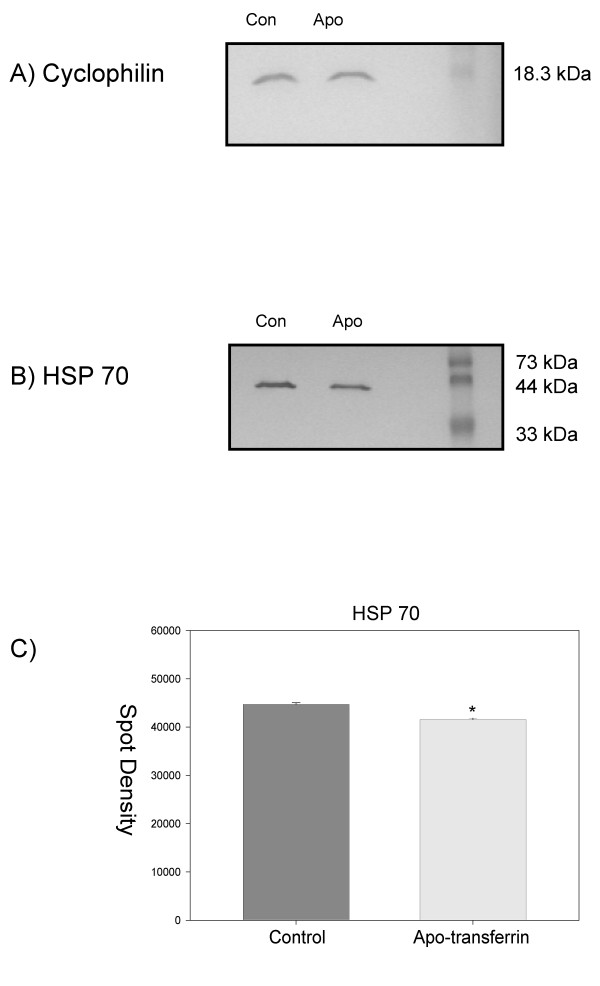
**Western blot analysis of proteins extracted from *H. capsulatum *grown with apo-tranferrin for 48 hr versus cultures free of apo-transferrin**. A) using antibody against Cyclophilin (S41, Figure 1) which showed no statistically different changes in protein abundance from our 2D gel analysis. B) using antibody against HSP70 which was found more abundant following 2D gel analysis in the medium free of apo-transferrin compared to medium containing apo-transferrin. C) Graphical representation of spot density calculation from biological replicates probing for HSP 70, p value = .034.

Western blot analysis was performed using antibodies specific to a protein that was not affected by iron levels as defined by 2D gel analysis, cyclophilin (Figure 4A), and a protein less abundant when iron levels were lowered, HSP 70 (Figure 4B). Statistical analysis of the calculated spot density values for HSP 70 biological replicates yielded a statistically significant difference, p value of .034 (Figure 4C). Differences between density values for cyclophilin were not significant.

## Methods

### Reagents

Trifluoroacetic acid (TFA) was obtained from Applied Biosystems (Warrington, WA). High performance liquid chromatography (HPLC)-grade acetonitrile, methanol, acetic acid, HPLC-grade water, ammonium bicarbonate, dithiothreitol (DTT), acrylamide, iodoacetamide, urea, and 3-[3-(Cholamidopropyl) dimethylammonio]-1-proanesulfonate (CHAPS) were obtained from Fisher (Fair Lawn, New Jersey). Carrier ampholytes (pH 3–10) and formic acid were purchased from Fluka Chemicals (Milwaukee, WI). SYPRO Ruby fluorescent dye was obtained from Invitrogen Molecular Probes (Carlsbad, CA). α-Cyano-4-hydroxycinnamic acid, bathophenathrolinedisulfonic acid (BPS), and protease inhibitor cocktail for fungal and yeast cells (P8215) was purchased from Sigma Aldrich (St. Louis, MO). Sequence grade trypsin was obtained from Promega (San Luis Obispo, CA). 4-chloro-1-naphthol HRP development was obtained from BioRad (Hercules, CA). HRP conjugate secondary antibodies were purchased from Pierce (Rockford, IL). HSP70 mouse monoclonal antibody was purchased from Sressgen (Victoria, BC Canada). Cyclophilin mouse monoclonal antibody was a gift from Francisco Gomez (University of Cincinnati, Infectious Disease).

### Sample preparation

*H. capsulatum *yeast (strain G217B) were prepared by inoculating 50 ml of medium, previously described [41], with 3 × 10^6 ^cells/ml from 3–5 days old slants to give a concentration of 1.5 × 10^8 ^cells/ml after 3 days. *H. capsulatum *yeasts were grown in medium set at pH 7.5 for 48 hr and switched to medium containing 5 μM apo-transferrin or medium free of apo-transferrin. One ml aliquots (~10^8 ^cells) were removed following 24 hr and 48 hr incubation with 5 μM apo-transferrin and medium free of apo-transferrin. Samples were then centrifuged at 1500 × g for 10 min. The supernatant was removed and 250 μl of lysis buffer (9 M urea, 2% CHAPS, 1% DTT, and 10 mM fungal and yeast cell protease inhibitor) was added. Cells were disrupted with a Min-Beadbeater (BioSpec Products, Bartlesville, OK) using an equal volume of glass beads for 30 sec and placed on ice for 30 sec. This was repeated 3 times. Following cell disruption, 250 μl of lysis buffer was added and samples were allowed to cool on ice for 10 min. Samples were then centrifuged at 15,000 × g for 30 min. An equal volume of water was added to each sample and further concentrated to 100 μl using an Amicon Ultra Centrifugal Filter Device. The clear supernatant was removed and protein concentrations were determined using the Bradford method.

We confirmed that apotransferrin did reduce iron levels by measuring the amount of iron bound to transferrin using the iron chelator bathophenathrolinedisulfonic acid (BPS) as described previously [42,43]. One ml of media containing 5 μM apo-transferrin was concentrated using Amicon Ultra Centrifugal Filter Device. To the concentrated transferrin (100 μl), nitric acid was added to give a 3% concentration followed by the addition of 38 mg/ml sodium ascorbate, 1.7 mg/ml BPS, and ammonium acetate (saturated ammonium acetate dilute 1/3) were added to the concentrated transferrin effectively denaturing the protein and reducing all iron to the ferrous form. After 5 min the absorbance was recorded at 535 nm for the concentrated retintate and flow through. Based on measuring the amount of iron bound to apo-transferrin the quantity of iron in the medium prior to addition of apotransferrin was .0008 g/L and following 24 hr in 5 μM apo-transferrin the concentration was reduced by ~83%. At 5 μM apo-transferrin changes in the amount of iron bound to apo-transferrin in the presence or absence of *H. capsulatum *was not detected.

Mass spectrometry was also used to measure the reduction of iron levels. The inductively coupled plasma mass spectrometer (ICP-MS) based quantification method has been commonly used to determine accurate concentration of metals in complex matrices. Specific iron detection in our samples was performed with an ICP-MS 7500cx (Agilent Technologies, Tokyo, Japan) equipped with an octopole collision/reaction cell system. Two isotopes of iron, ^56^Fe^+ ^and ^57^Fe^+^, were monitored using He as collision gas at 4.0 ml min^-1 ^to reduce the ^40^Ar^16^O^+ ^and ^40^Ar^16^O^1^H^+ ^interferences. The RF power was 1450 W and carrier gas flow rate was 1.02 L min^-1^. Apo-transferrin medium was filtered through a 10 kD membrane filter before analyzed by ICP-MS. Total iron levels were lowered from 57.15 ppb (± .84), to 5.93 (± 1.27) ppb when 5 μM of apo-transferrin was added to the medium, a reduction of ~90% of the total iron. Standard deviations were calculated from 3 biological replicates.   

### 2D-gel electrophoresis and statistical analysis

Fifty μg of protein were mixed with rehydration buffer (9 M urea, 2% CHAPS, 1% dithiothreitol (DTT), and carrier ampholyte (pH 3–10) solution, 20 μl/1 ml). A total of 350 μl of this rehydration solution was loaded onto 24 cm immobilized protein gradient (IPG) strips pH 3–10 (Amersham Biosciences, GE Healthcare, Piscataway, NJ) and incubated (rehydrated) for 12 hr. Isoelectric focusing (IEF) was performed with an IPGphor system (Amersham Biosciences) using a step gradient starting from 0–500 V for 1 hr, 500–1000 V 1 hr, 1000–8000 V 12.5 hr, and 8000V for 2.5 hr. Following IEF, strips were stored at -70°C.

Strips were then equilibrated for 30 min in 15 ml of 6 M urea, 20% glycerol, .05 M Tris, 2% SDS, 75 mg DTT, and a few crystals of bromophenol blue. Strips were then loaded onto a 10% SDS PAGE gel. The second dimension was run at 200 V for 15 min followed by 300 V for 4–5 hr. Gels were next fixed for 15 minutes in 10% methanol/7% acetic acid. Gels were then stained overnight with SYPRO Ruby fluorescent dye and destained for 20 minutes in fixing solution. Gel images were produced using a GE Healthcare Typhoon scanner. Before image analysis, gels were washed with water for 5–10 min.

2D gel spot detection, matching, and spot volume, calculations were performed using SameSpots (Nonlinear Dynamics, Durham, NC) software on three biological replicates, six gels per 24 and 48 hr apo-transferrin treatments and their respective controls. Spot volumes for all protein spot matches within each analysis were calculated and normalized to the total spot volume yielding a spot volume ratio. This ratio enabled the calculation of average abundance changes across all 3 replicates within each test group and the application of ANOVA statistical test. Individual spots were ranked by p-value from a one way global ANOVA analysis, with maximum fold change based on spot normalized volume. P values of < 0.05 were considered statistically significant and reflect the probability that the observed change did not occur from random noise.

### H. capsulatum viability test

To verify that the proteins found altered in our analysis did not result from the death of *H. capsulatum*, the organism was plated on solid medium following 24 and 48 hr growth with apo-transferrin. One hundred yeast were plated from cultures growing with or without apo-transferrin for 48 h. Eighty four colonies were counted on plates from *H. capsulatum *cultures containing apo-transferrin while 82 were counted from cultures free of apo-transferrin. Thus any changes we might find would be a direct response from the stress we were applying, and not from *H. capsulatum *death.

### In-gel digestion

Protein bands were excised and diced into small pieces (~1 mm) and placed into 0.65 mL siliconized tubes (PGC Scientific). Each gel piece was briefly incubated and washed with 100 μl of water. Gel pieces were resuspended in 100 μl of acetonitrile and placed in a speed vacuum until dry. Next, 30 μL of 10 mM DTT in 25 mM ammonium bicarbonate was added to the dried gel pieces. Samples were vortexed and centrifuged briefly. The reaction was allowed to proceed at 56°C for 1 hr. The supernatant was removed and 30 μl of 55 mM iodoacetamide was added to the gel pieces. Samples were vortexed and centrifuged briefly. The reaction was allowed to proceed in the dark for 45 min at room temperature. Supernatant was removed and the gels were washed with 100 μl of ammonium bicarbonate by vortexing for 10 min. Supernatant was removed and gel pieces were dehydrated in 100 μL of 25 mM ammonium bicarbonate/50% ACN, vortexed for 5 min, and centrifuged. This step was repeated. Gel pieces were placed in a vacuum centrifuge until dry. Thirty microliters of a 12.5 ng/ml trypsin solution was added to the gel pieces and rehydrated on ice at 4°C for 10 min. Supernatant was removed, samples were centrifuged, and 25 μL of 25 mM of ammonium bicarbonate was added. Samples were centrifuged briefly and incubated at 37°C overnight. Following overnight digestion the solution was placed into a clean 0.65 mL siliconized tube. To the gel pieces, 50 μL of 50% acetonitrile/5% trifluoroacetic acid acid (TFA) was added. Samples were vortexed for 30 min, sonicated for 5 min, and the solution was combined with the first aqueous extraction. This was repeated and samples were then dried in a vacuum centrifuge.

### MALDI-TOF mass spectrometric analysis

Dried peptide extracts were dissolved in 5 μL of water. Next, 2.5 μL of sample was mixed with 2.5 μL of alpha-cyano-4-hydroxycinammic acid in 50% acetonitrile/5% TFA, 10 mg/mL, and 1 μL of sample was delivered to a gold plated matrix assisted laser desorption/ionization time-of-flight (MALDI-TOF) mass spectrometer (MS) sample plate. Mass spectra were collected on a Voyager-DE PRO (Applied Biosystems, Foster City, CA) MALDI-TOF MS. Each spectrum was an average of 200 laser shots. Trypsin autodigestion peaks (2211.11 and 842.51) were used to calibrate each spectrum.

Raw MALDI-TOF data were processed using manufacturer-supplied Data Explorer software, with the following settings: Baseline correction parameters; peak width 32, flexibility .5, and degree .1 Noise filter/smooth was selected with a correlation factor of .7. Each MALDI spectrum was manually inspected to create a peak list. To control for peaks generated from keratin and trypsin, a spot was cut from an area of the gel containing no protein, and prepared with each sample. Peaks which were in both keratin/trypsin control and sample spectra were excluded from peak lists.

### LC-MS/MS analysis

For nanoflow liquid chromatography (LC)-MS/MS analysis, dried peptide extracts were dissolved in 5 μL of 2% acetonitrile, 0.1% formic acid in water. Resuspended samples were loaded onto a Symmetry 5 μm particle, 180 μm × 20 mm C18 precolumn (Waters, Milford, MA), then washed 5 min with 1% acetonitrile in 0.1% formic acid at a flow rate of 20 μL/min. After washing, peptides were eluted and passed through an Atlantis 3 μm particle, 75 μm × 100 mm C18 analytical column (Waters, Milford, MA) with a gradient of 1–80% Acetonitrile in 0.1% formic acid. The gradient was delivered over 120 min by a nanoACQUITY UPLC (Waters, Milford, MA) at a flow rate of 250 nl/min, to a fused silica distal end-coated tip nano-electrospray needle (New Objective, Woburn, MA). Data were collected on a Q-TOF Premiere mass spectrometer (Waters, Milford, MA) set for MS survey scans and automatic data-dependent MS/MS acquisitions, which were invoked after selected ions met preset parameters of minimum signal intensity of 10 counts per second, ion charge state 2+, 3+, or 4+, and appropriate retention time. Survey scans of 1 s were followed by collision induced dissociation (CID) of the three most intense ions for up to 6 s each, or until 10,000 total MS/MS ion counts per precursor peptide were attained.

Raw LC-MS/MS data were processed using manufacturer-supplied ProteinLynxGlobalServer 2.2 software, with the following settings: Adaptive background subtraction of polynomial order 5 below a 35% curve, two smooths with a window of three channels in Savitzky Golay mode, followed by fast deisotoping and centroid calculation of the top 80% of peaks based on a minimum peak width of 4 channels at half-height. On the basis of these parameters, pkl files incorporating parent ion mass and peak lists for each corresponding MS/MS spectrum were generated.

### Database searching

Protein/genomic databases 1, 2 and 3 were constructed beginning with the *H. capsulatum *G217B strain genome obtained from Washington University's Genome Sequencing Center website . Database 1 was constructed using only Fgenesh and parameters setup by Softberry . Database 2 was assembled using publicly available EST libraries, also retrieved from Washington University's Genome Sequencing Center website . EST's were generated from *H. capsulatum *cDNA under both mycelial and yeast phases, and assembled into full gene sequences with the aid of the contig assembly program (CAP)3 genome assembly program [29]. Database 3 was assembled using a combination of the gene finding programs Fgenesh, EAnnot, GeneWise, and SNAP [28,30-32].

MALDI-TOF MS and LC-MS/MS data were used to search in-house databases 1–3 and publicly available fungal databases using MASCOT (version 2.1, Matrix Science, London, United Kingdom). A protein match from either of the 3 databases would count as a hit. Only protein matches deemed statistically significant (p < .05) by the MASCOT algorithm were accepted as protein sequence matches. For MALDI-TOF data, a 50 ppm mass accuracy threshold was set while a minimum precursor and fragment-ion mass accuracy of 1.2 and 0.8 Daltons was required for LC-MS/MS data. Up to 1 missed trypsin cleavage was allowed while fixed and variable carbamidomethyl were selected for separate searches. A decoy database was constructed by scrambling our in-house database sequences, and used to estimate false positive protein ID rates. Searching MS data against the decoy database did not result in any significant MASCOT scores. In order to annotate the protein sequence matches obtained from searching databases 1–3, each sequence was BLAST searched against the NCBInr sequence database.

### Western blot analysis

*H. capsulatum *proteins prepared for 2D gel analysis were also probed using different antibodies specific for either cyclophilin or HSP 70. Ten micrograms of protein was mixed with 1× sample buffer (5×; 200 mM Tris-HCl, pH 6.8, 50% glycerol, 5% SDS, .5% bromphenol blue, and 5% beta-mercaptoethanol) heated at 95°C, and loaded onto a 12% SDS PAGE gel. Gels were run at 150 V for ~2 hrs. Following electrophoresis, gels were incubated in transfer buffer (25 mM Tris, 192 nM glycine, 15% v/v methanol) for 15 min. Proteins were transferred from 1D gels to nitrocellulose membrane overnight at 30V and 60V for 1 hr. Following transfer, each membrane was placed in 20 ml of blocking buffer for 1–2 hr. Blocking buffer consisted of 1× Tris-buffered saline (TBS) 5 g of non-fat dried milk/2.5 ml of 10% Tween. 10× TBS consisted of 24.2 g Tris base, 80 g of NaCl, pH 7.6. Primary antibody was added in a 1/5000 (v/v) ratio of antibody to blocking buffer and the membranes were incubated overnight at 4°C. This was followed by five 5 min washes in blocking buffer. Secondary horseradish peroxidase (HRP) conjugated antibody was added in a 1/1000 ratio of antibody to blocking buffer and the membranes were incubated for 1 hr. Membranes were then washed 5 times in 1× TBS for 5 min each. Immunoreactive bands were visualized using BioRad HRP color development reagent 4-chloro-1-naphthol and the AlphaImager 950 high-performance CCD camera. Spot densities were quantified using AlphaImager software.

### 2D gel analysis of H. capsulatum isolated from IFN-γactivated macrophages

Bone marrow derived macrophages were harvested 5 days following incubation with 10 ng/ml granulocyte macrophage-colony stimulating factor (GM-CSF). Macrophages were incubated for 24 hr with 5 ng/ml of IFN-γ. Leucine uptakes assays, as previously described^7^, showed that macrophages were activated to inhibit *H. capsulatum *growth by 80% (data not shown). Macrophages were infected with *H. capsulatum *at a multiplicity infection of 5 for 24 hrs. Following 24 hr incubation, macrophages were washed to remove any *H. capsulatum *that had not been ingested. Macrophages were then lysed in a buffer containing 19%, ethanol, .1% SDS, and 1% phenol. An *H. capsulatum *control was also prepared under the exact conditions except for the addition of macrophages. Cells were centrifuged at 2000 G's and the supernatant containing macrophage debris was removed. *H. capsulatum *cells were washed, lysed, and 2D gel analysis was performed as previously described. In order to retrieve an adequate amount of *H. capsulatum *protein approximately 3.0 × 10^7 ^macrophages needed to be infected for one biological replicate.

## Conclusion

Using a proteomic approach we have begun to address the knowledge gap which exists regarding the identities and number of proteins regulated by *H. capsulatum *under host conditions. Among the *H. capsulatum *proteins whose regulation was influenced by lowering iron availability were metabolic enzymes involved in glycolysis, lysine biosynthesis, and the TCA cycle. Like *S. cerevisiae *and *C. ablicans *[37,40], *H. capsulatum *may adjust its metabolism to more efficiently use the iron that is available. This modulation might be advantageous for survival in a nutritionally restricted environment. Recent studies have shown ferrous, but not ferric, iron maintains homoeostasis in *H. capsulatum *triacylglycerides indicating a role for lipid metabolism in relation to iron [27].

A shift in metabolism and a 2.0% reduction in protein abundance suggest that the organism is reducing the activity of expendable cellular processes to cope with environmental changes similar to those encountered in the host. Three of the *H. capsulatum *enzymes altered by iron levels; asparaginyl-tRNA synthetase, saccharopine dehydrogenase, and a protein with an amino acid sequence unique to *H. capsulatum *have yet to be connected to pathogen iron availability thus warranting further investigation.

## Competing interests

The authors declare that they have no competing interests.

## Authors' contributions

MSW designed the experiments, analyzed and interpreted data, and prepared the manuscript. DSS helped with LC-MS/MS analysis, MS data interpretation, and manuscript preparation. FJG and AGS contributed by helping construct the *H. capsulatum *genomic/proteomic database. MH helped by performing 2D gel electrophoresis experiments. QC and BC provided ICP-MS analysis. QC also helped with data interpretation and manuscript preparation. TAN and GSD contributed to the preparation of manuscript. All authors read and approved the final manuscript.

## Supplementary Material

Additional file 1 Fold changes and p values from the 42 protein spots altered in abundance following *H. capsulatum *growth in media containing 5 μM apo-transferrin for 24 and 48 hr. List of p values and fold changes for all of the *H. capsulatum *proteins found differentially expressed when iron levels were lowered. Click here for file

Additional file 2 *H. capsulatum *malate dehydrogenase protein expression following ingestion by macrophages. Magnified region of *H. capsulatum *malate dehydrogenase, spot 17 (A31) from 2D gel analysis of *H. capsulatum *A.) grown in the presence of a macrophage lysis buffer B.) following isolation from IFN-γ activated bone marrow derived macrophages. Protein band outlines were created by the SameSpots software. Each biological replicate is labeled 1, 2, or 3. The MALDI-TOF and bioinformatic analysis of the in-gel digestion of this protein spot yielded a MASCOT score of 157 with 9/10 masses matched covering 45% of the of the malate dehydrogenase protein sequence. Click here for file
