# Supplementary figures and images for: Histoplasma capsulatum proteome response to decreased iron availability
Source: Proteome Sci. 2008 Dec 24;6:36. doi: 10.1186/1477-5956-6-36 (PMC2645362; doi:10.1186/1477-5956-6-36)

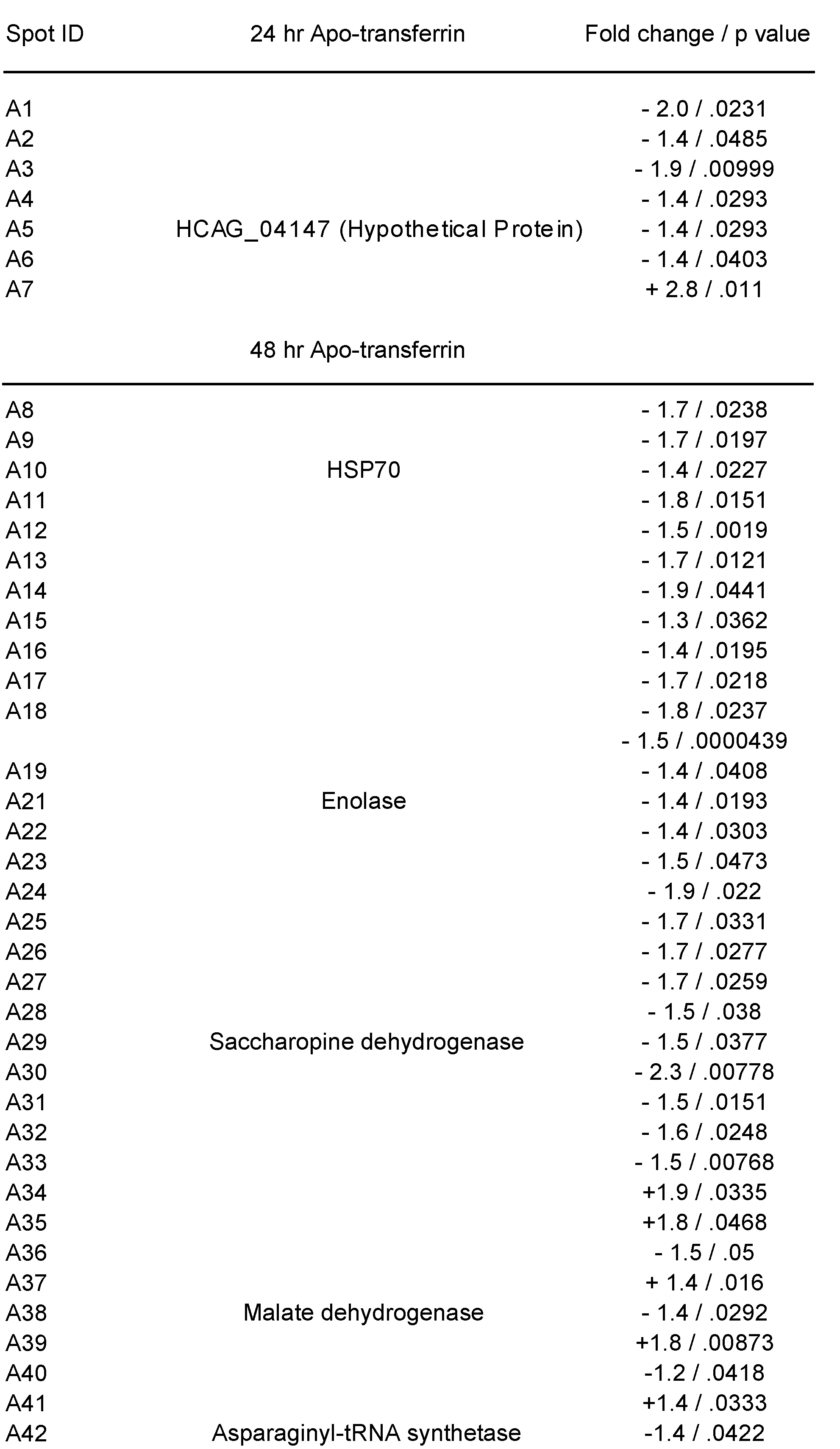

Supplement: Additional file 1 — Fold changes and p values from the 42 protein spots altered in abundance following H. capsulatum growth in media containing 5 μM apo-transferrin for 24 and 48 hr. List of p values and fold changes for all of the H. capsulatum proteins found differentially expressed when iron levels were lowered. [file 1477-5956-6-36-S1.tiff]

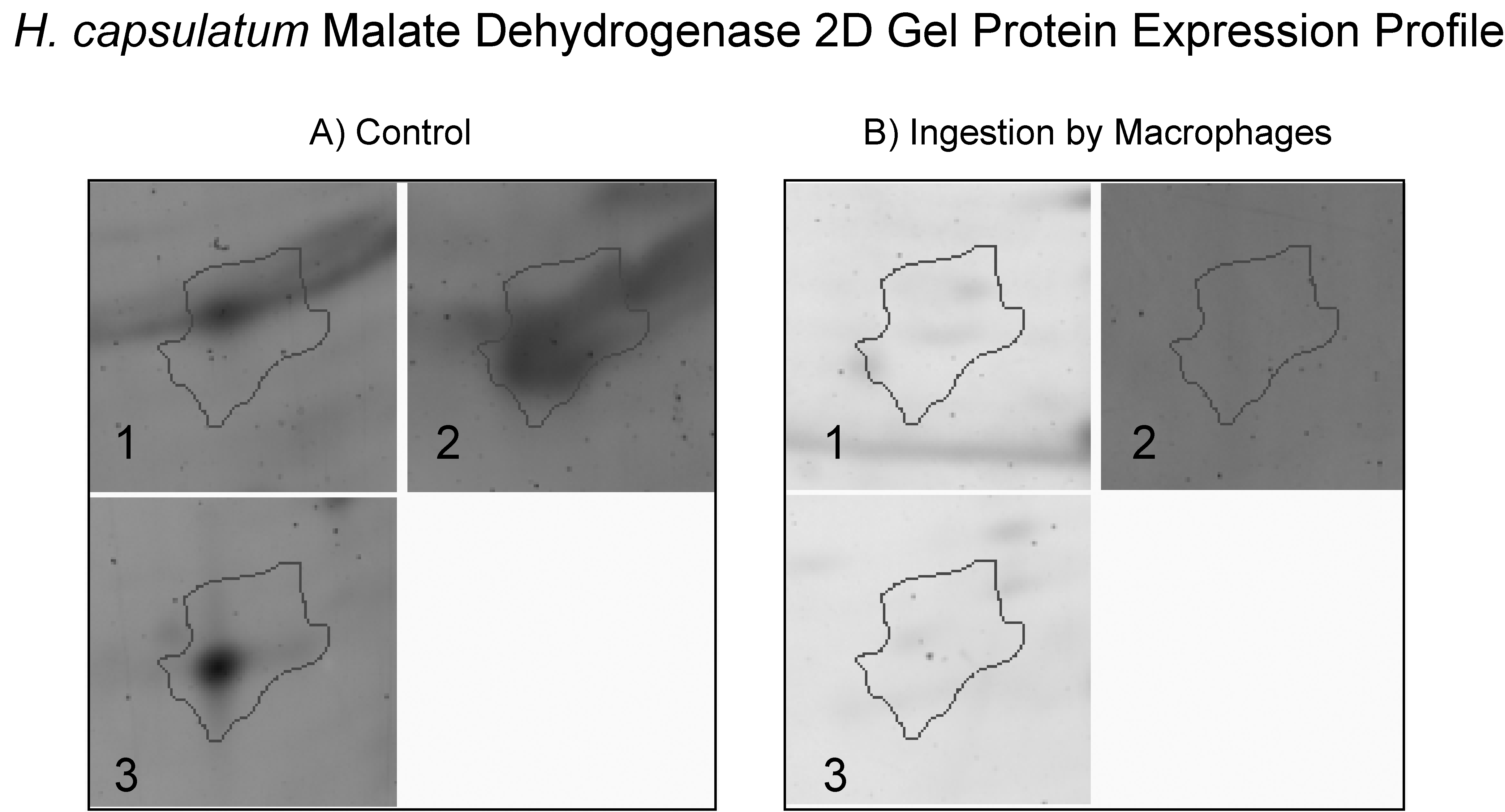

Supplement: Additional file 2 — H. capsulatum malate dehydrogenase protein expression following ingestion by macrophages. Magnified region of H. capsulatum malate dehydrogenase, spot 17 (A31) from 2D gel analysis of H. capsulatum A.) grown in the presence of a macrophage lysis buffer B.) following isolation from IFN-γ activated bone marrow derived macrophages. Protein band outlines were created by the SameSpots software. Each biological replicate is labeled 1, 2, or 3. The MALDI-TOF and bioinformatic analysis of the in-gel digestion of this protein spot yielded a MASCOT score of 157 with 9/10 masses matched covering 45% of the of the malate dehydrogenase protein sequence. [file 1477-5956-6-36-S2.tiff]
